# Impact of microchannel width on axons for brain-on-chip applications†

**DOI:** 10.1039/d4lc00440j

**Published:** 2024-10-23

**Authors:** Katarina Vulić, Giulia Amos, Tobias Ruff, Revan Kasm, Stephan J. Ihle, Joël Küchler, János Vörös, Sean Weaver

**Affiliations:** a Laboratory of Biosensors and Bioelectronics (LBB), ETH Zürich 8092 Zürich Switzerland voros@ethz.ch sean.michael.weaver@alumni.ethz.ch

## Abstract

Technologies for axon guidance for *in vitro* disease models and bottom up investigations are increasingly being used in neuroscience research. One of the most prevalent patterning methods is using polydimethylsiloxane (PDMS) microstructures due to compatibility with microscopy and electrophysiology which enables systematic tracking of axon development with precision and efficiency. Previous investigations of these guidance platforms have noted axons tend to follow edges and avoid sharp turns; however, the specific impact of spatial constraints remains only partially explored. We investigated the influence of microchannel width beyond a constriction point, as well as the number of available microchannels, on axon growth dynamics. Further, by manipulating the size of micron/submicron-sized PDMS tunnels we investigated the space restriction that prevents growth cone penetration showing that restrictions smaller than 350 nm were sufficient to exclude axons. This research offers insights into the interplay of spatial constraints, axon development, and neural behavior. The findings are important for designing *in vitro* platforms and *in vivo* neural interfaces for both fundamental neuroscience and translational applications in rapidly evolving neural implant technologies.

## Abstract 2

Axon growth is influenced by the width and number of microchannels, with growth cone adaptability reaching its limit for constraints smaller than 350 nm. Tradeoffs between axon growth and control need to be made for neurofluidic devices.

## Graphical abstract

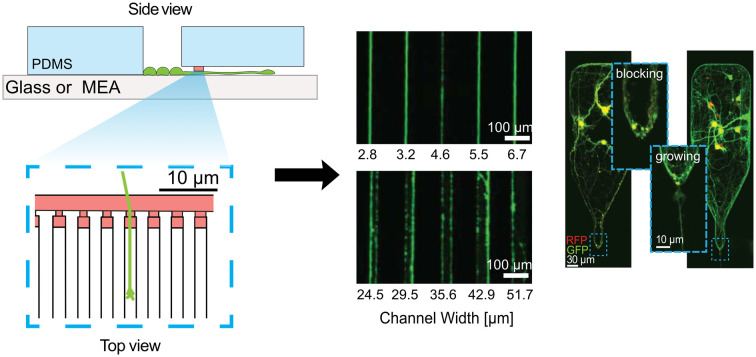

## 1 Introduction

Neurons are the building blocks of the nervous system having a unique structure and polarity^1–3^ essential for efficient information transmission. The polarity of neurons is expressed by two distinct subcellular components: dendrites and axons. Dendrites are projections emerging from one side of the cell soma, acting as the main computational units of the neuron.^4^ They receive information from multiple presynaptic partners through synapses which can trigger action potentials (AP) to propagate through axons. AP elicitation and subsequent propagation are the primary form of communication between neurons.^5,6^ Axon growth is vital for functional development and potential repair of the nervous system. It relies on an interplay between extracellular and intracellular cues,^7^ whose dynamics are still not fully understood. Given the challenges studying the complex central nervous system *in vivo*,^8–11^ isolated mechanisms of axon growth have been extensively studied *in vitro*.^1,12–15^ A substantial amount of research has been done with rat primary cortical,^11,16^ thalamic^11,17^ and hippocampal^18^ neurons. For these cells, several stages of growth and development have been studied and defined. Within hours of seeding, neurons undergo morphological changes from rounded symmetrical cells to polar cells with distinct dendrites and axons. Over the next days *in vitro*, the axon growth cone, a mobile and flexible sensory structure at the axon tip, explores its environment while receiving guidance cues from the extracellular matrix and neighboring axons to enforce the direction and extent of axon growth.^19^ The tension forces between an axon and a substrate,^20^ and forces between axons (axon fasciculation^21,22^ orchestrated by the growth cone^23^) play an important role in determining their growth extent and trajectory. Namely, axon bundles constitute of two types of axons, *i.e.* pioneers and followers.^22^ Pioneer axons are like explorers, showing a stop-and-go pattern with alternating periods of movement and rest. In contrast, follower axons grow faster and more steadily, with fewer pauses along their path. Understanding and leveraging these mechanisms is crucial not only for understanding nervous system development but also for exploring the potential of axon regeneration for advancing therapeutic interventions in cases of nerve damage.

Studying neurite outgrowth in random cultures hinders our understanding of the development of individual axons and the factors governing axon length. For instance, distinguishing axons from axon bundles is challenging due to inherent axon size variability^17,24^ hampering the tracking of unique developmental trajectories. Differences in development between isolated axons and axon bundles further complicate the analysis. Additionally, assessing the impact of extracellular cues on growth cone exploration or accurately measuring axon length face complications in the absence of a controlled environment. To establish more controllable and reproducible experimental systems, researchers have increasingly turned to controlling neural network topology through patterning. Some methods applied to control the network topology *in vitro* have been inspired by *in vivo* studies that demonstrate directional guidance through adhesion forces.^25^ The aforementioned interactions of axons with a substrate and axon fasciculation are both critical to consider in the design of culture tools for axon guidance.^26^ By providing controlled surfaces with cell-adhesive coatings or adjusting the mechanical properties of substrates, researchers can guide axon growth and explore the interplay between neurons and their environment.^27^ The substrate can be modified by techniques such as microcontact printing,^16,20^ photolithography,^28,29^ electrochemical surface modification,^30^ and light-induced nanotopography^31^ in order to manipulate network topology. Notably, patterning methodologies can be combined with microelectrode arrays (MEAs),^30,32,33^ allowing for the recording and modulation of extracellular neural activity, thus providing a functional aspect to create engineered neural systems. This way neural development can also be monitored in terms of functionality in parallel with its morphology.

Despite the benefits from the described patterning methods, there remain challenges to be addressed. Coatings may degrade over time or be influenced by axon forces, limiting their suitability for extended culturing.^27,34,35^ Achieving precise neurite guidance^36^ and controlling neural network size^27,37^ remains challenging. To address these concerns, an alternate patterning approach involves physical confinement through polydimethylsiloxane (PDMS) microstructures. PDMS microstructures are biocompatible and gas-permeable, hence they support long-term cell culturing.^37^ Their transparency facilitates high-resolution imaging, and compatibility with MEAs allows for integrated functional analysis.^38^ Traditionally, PDMS microstructures with dual parallel culture chambers have been widely utilized for co-culturing different cell types and targeted drug delivery, as highlighted by Taylor *et al.*^39^ However, the design of PDMS microstructures offers many possibilities beyond this standard approach. Recent PDMS designs can achieve subcellular compartmentalization and precise control over directionality, as demonstrated in recent studies.^40–45^ Researchers can customize the network size and topology of PDMS microstructures to suit specific experimental needs. This flexibility provides a powerful platform for investigating neural dynamics and other complex cellular phenomena.

In the context of axon development, PDMS microstructures offer an opportunity to investigate spatial limitations of axon growth. It has been observed that axons tend to follow edges and avoid turning in more than 90 degree angle.^40,41,46,47^ The microchannel height influence on electrophysiology recording fidelity has been systematically addressed using two-compartment microfluidics systems.^48^ However, the effects of spatial constraints in terms of width of the microchannels on axon growth, specifically in the lower, submicron range has not yet been studied in detail. For example, it is still unknown how constraints affect the extent of axon bundle growth, how sensitive conduction speed of axon bundles is to axon bundle size and if spatial constraint modifies the speed. Furthermore, growth cone adaptability to spatial constraints is not fully explored. How they regulate their length according to their width, for instance on a surface considered as effectively infinite along one direction, has not been quantitatively addressed in detail.^49^ The morphological adaptability of growth cones, spanning dimensions from a few microns to several tens of microns,^1,50–52^ empowers axons to dynamically adjust to the diverse extracellular spaces they encounter. This adaptability is particularly important in navigating divergent extracellular environments found *in vivo*. Moreover, it has been shown that the size and morphology of growth cones can indicate the functionality of the particular axon, distinguishing between the aforementioned pioneer and follower axons,^22,53,54^ presenting yet another important role of growth cones in axon development.

In this work we investigate how variations in the number of efferent channels and channel size of PDMS microstructures impacts axon growth rate, bundle formation, and activity propagation. By reducing the size of the topological restriction to the same order or smaller than the growth cone we study the morphological adaptability of the growth cone by identifying the narrowest space restriction axons can penetrate.

## 2 Materials and methods

### 2.1 PDMS microstructures

**Fig. 1:**
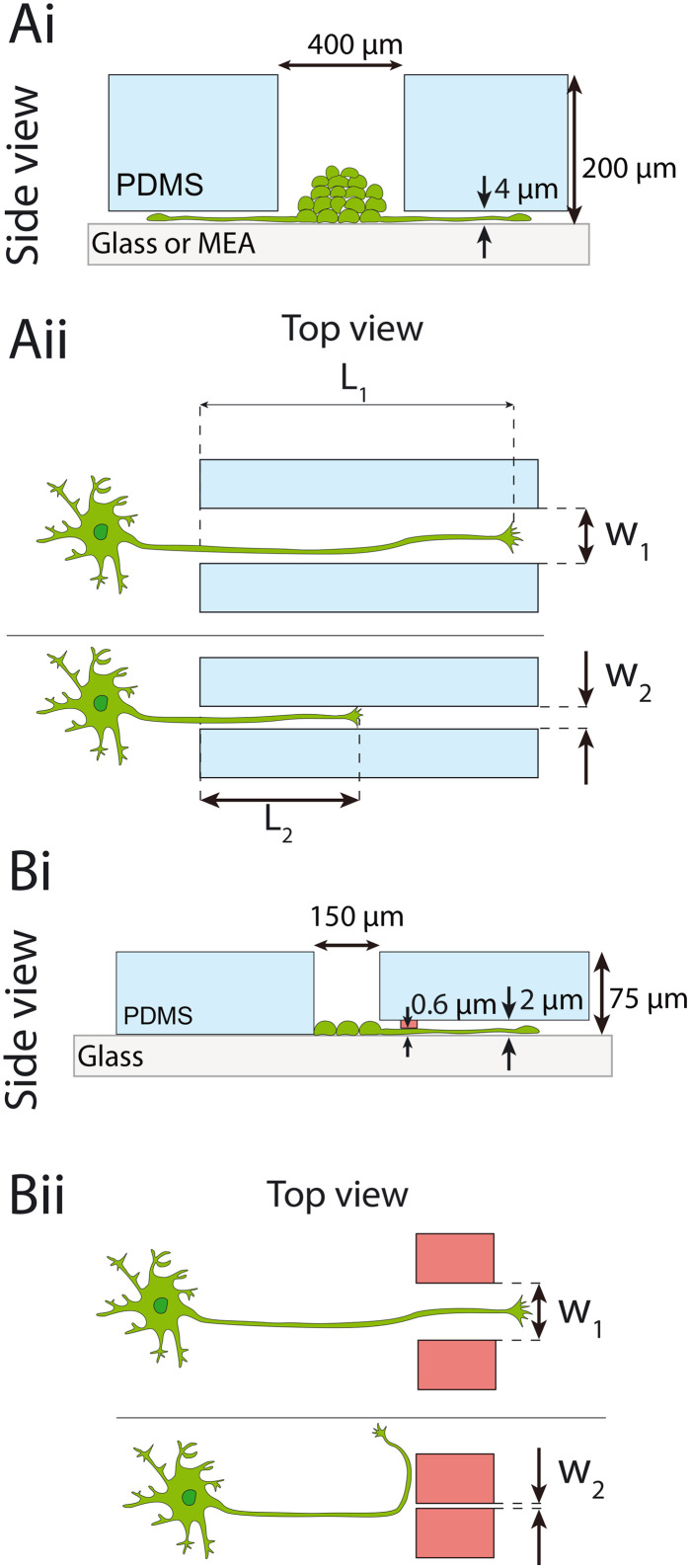
Overview of the PDMS microstructures used in this study for spatially confining axon growth. Ai) side view of the spheroid-seeding microstructure. Spheroids are seeded into the wells. Microchannels that emerge from the wells are too narrow for the cell soma to grow through but allow for axon growth. Aii) The length of axons, starting from the beginning of the microchannel, was investigated for different microchannel widths. Bi) Side view of the thin microstructure. Low-density cell suspension is seeded into the wells. Microchannels contain additional submicron constraint (depicted in red). Bii) The spatial limitation of axon growth is assessed by noting what is the smallest constraint a growth cone can penetrate.

PDMS microstructures for cell and axon guidance were designed in a CAD software (AutoCAD 2021) and fabricated by Wunderlichips (Zurich, Switzerland). The fabrication process is described in former publications.^41,43^ All microstructures used in this study have wells where the cells are seeded and microchannels which are impermeable for soma but accessible for neurites. Schematics of the microstructures are shown in Fig. 1. The PDMS thickness and microchannel height vary between structure types. 200 μm thick microstructures will be referred to as spheroid-seeding PDMS microstructures and 75 μm thick microstructures as cell-suspension-seeding PDMS microstructures. The height difference between the two PDMS microstructure types arises from small variations in their microfabrication processes. The second set of microstructures necessitates a modified protocol from the standard fabrication method to successfully produce submicron features, as detailed in the earlier work by Mateus J. C. *et al.*^43^

#### 2.1.1 Spheroid-seeding PDMS microstructures

**Fig. 2:**
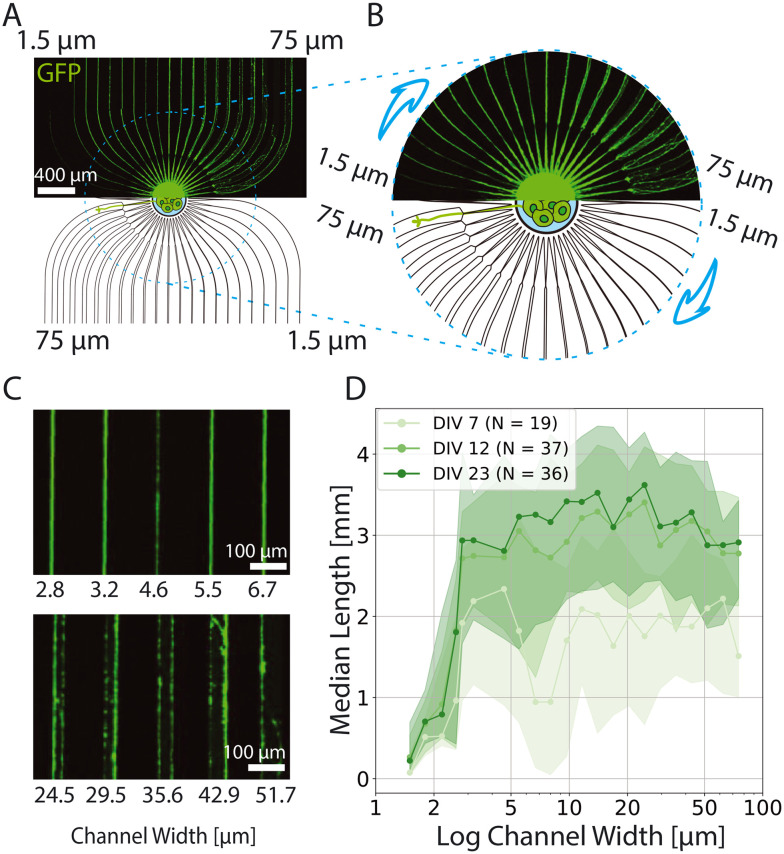
Spheroid-seeding PDMS microstructure for studying the length of axons for different microchannel widths. A) Overview of the microstructure. The bottom half shows the schematic of the microstructure, while the upper half shows neurons expressing GFP at DIV 12. B) Close up image of the seeding well and microchannels emerging from it. All microchannels initially narrow down to 1.5 μm in width and then extend to the final microchannel size that increases from 1.5 to 75 μm as indicated by an arrow. C) Example of axon growth through various microchannel widths. D) Median axon length with the corresponding quartile range indicated as shading as a function of channel width for different DIV. Note that *x* axis is represented by a logarithmic scale. Number of biological replicates is 6.

**Fig. 3:**
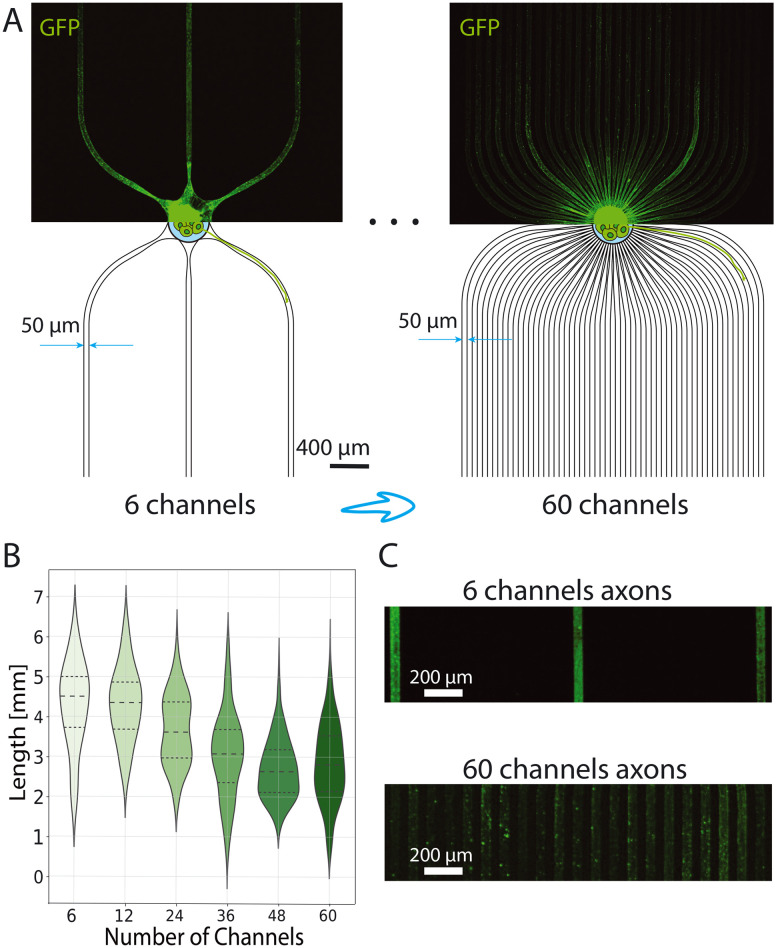
Controlling the number of axons per microchannels with spheroid-seeding PDMS microstructures with different number of microchannels emerging from the central well. A) The two extreme examples of the microstructures. The bottom half shows the schematics and the dimensions of the microstructures, while the upper half shows GFP-expressing neurons at DIV 23. The number of microchannels was varied from 6 to 60. B) Example of axons growing in the 6-channel (top) and 60-channel(bottom) microstructure. C) Violin plot with a corresponding quartile range for the axon length dependency on the number of microchannels emerging from the central well. The number of technical replicates is 7, 8, 10, 8, 8, 7 in the ascending order of the microchannel number.

**Fig. 4:**
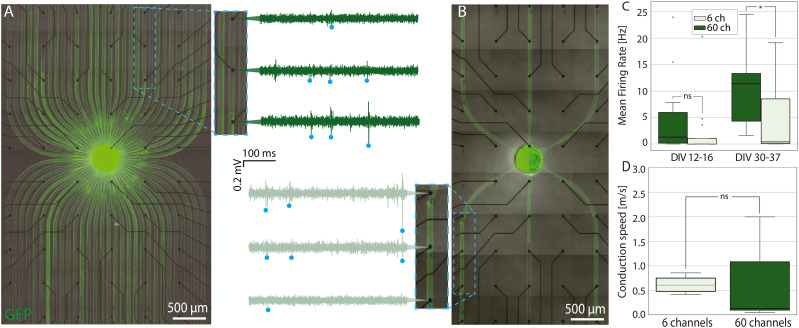
Spheroid-seeding PDMS microstructures for studying the electrophysiology of bundled axons. A) 60-channel PDMS microstructure aligned on top of a transparent 6 × 10 MEA and the corresponding voltage traces recorded from three selected electrodes along the same channel. B) 6-channel PDMS microstructure on top of a MEA and the corresponding voltage traces recorded from electrodes along the same channel. C) Mean firing rate recorded and calculated in the DIV range 12–16 and 30–37 for the cells in 6- and 60-channel PDMS microstructure respectively. A star corresponds to a *p*-value < 0.05. Kruskal-Wallis test was performed to evaluate differences. D) AP conduction speed calculated using the delay times derived from spike-time triggered histograms in 6- and 60-channel PDMS microstructures. Number of biological replicates is 2.

The PDMS microstructures used in the experiments investigating the effect of channel width in Fig. 2 and channel number in Fig. 3 and 4 are 200 μm thick and consist of a 400 μm diameter well to accommodate neural spheroids. The well then branches into microchannels. Each microchannel is 4 μm high along the whole length. The 4 μm height of the microchannels makes it impermeable for cell soma but accessible for axons (and dendrites). Microchannels are 8 mm long. Schematics can be found in Fig. S1 and S2.† The PDMS microstructure shown in Fig. 2 have 44 concentric microchannels emerging from the central seeding well that narrow down to 1.5 μm in width to provide similar starting conditions. After the 1.5 μm wide constriction, these channels branch into microchannels with widths varying from 1.5 to 75 μm. The design has a point symmetry so each half is considered as a separate replicate. The PDMS microstructure shown in Fig. 3 has microchannels with the identical width of 50 μm. The radial microstructures with single central well were chosen as opposed to the standard two-compartment microstructures to facilitate measuring the extent of bundle growth. Namely, radial symmetry removes the potential bias that might be caused by axons preferring one channel over another due to the location of the spheroid inside the well.

#### 2.1.2 Cell-suspension-seeding microstructures with variable submicron tunnel width

The thickness of the PDMS microstructures shown in Fig. S3 and S4† is 75 μm and it consists of eight wells of area 120 × 260 μm^2^ that are suitable for seeding cells in suspension. They feature 2 μm high microchannels with submicron size, 600 nm high tunnels. In the case of microstructures shown in Fig. S3,† seven seeding wells narrow down to nano/submicron channel tunnels with a height of 600 nm and width varying from 0.6 to 1.8 μm which then extend to a width of 30 μm. The eighth channel has a fixed width of 30 μm and serves as a control. The PDMS microstructure described in Fig. S4† consists of a hexagonal seeding well with a 145 μm long side that narrows down to thirteen tunnels with widths varying from 150 to 1000 nm. The microchannels that follow are 0.65 mm long.

### 2.2 Substrate preparation

#### 2.2.1 Glass bottom dish

Culture dishes with microscopy glass bottom were used for imaging experiments. 30 mm diameter glass coverslips (Menzel glass, selected no. 1.5, ThermoFisher) were mounted to plastic rings (WillCo Wells) according to the manufacturer's instructions. The glass bottom thickness is 170 μm, which makes it suitable for high resolution imaging. Mounted dishes were filled with isopropanol, ultrasonicated for 10 min and afterwards further rinsed with isopropanol, and ultrapure water (Milli-Q, Merck-MilliPore) before being blow-dried with nitrogen.

Next, the dishes were treated with air plasma for 2 min (18 W PDC-32G, Harrick Plasma) and coated with 500 μL per dish of 0.1 mg mL^−1^ poly-d-lysine (PDL) (P6407, Sigma Aldrich) in phosphate buffered saline (PBS) (10010-023, ThermoFisher). After PDL coating at room temperature for 45 min, dishes were rinsed three times with ultrapure water. Ultrapure water was then aspirated and the dish was carefully blow-dried with nitrogen.

#### 2.2.2 Microelectrode array

Glass MEAs (60MEA500/30iR-Ti-gr, Multichannel Systems) with a 6 × 10 electrode grid, electrode spacing of 500 μm, and an electrode diameter of 30 μm were used for experiments requiring extracellular activity recordings. MEAs were reused across several experiments. When reused, MEAs were rinsed with ultrapure water and immersed in 4% Tergazyme (1304-1, alconox) overnight to remove organic debris. MEAs were immersed in ultrapure water and placed in the fridge for long-term storage. Prior to cell seeding, MEAs were rinsed with isopropanol and ultrapure water and dried with nitrogen. After thorough drying, MEAs were treated with air plasma for 2 min and coated with 500 μL per MEA of 0.1 mg mL^−1^ PDL for 45 min at room temperature. MEAs were afterwards rinsed with ultrapure water and carefully blow-dried with nitrogen.

#### 2.2.3 Microstructure attachment

The PDMS membrane was cut with a scalpel and an individual microstructure was placed on the substrate with tweezers. For MEAs, the microchannels were aligned along the electrodes under a light microscope (Leica Microsystems, Germany) using a drop of ultrapure water and tweezers. Upon placing the microstructure on the substrate and subsequent visual inspection the dishes were left in the oven at 37 °C for 45 min. Afterwards, PBS was added in the dishes and they were placed in the desiccator for 10 min or until there were no air bubbles coming out of the microstructures. After desiccation, PBS was exchanged with NeuroBasal (NB) medium and the dishes were ready for cell seeding. The thinness of the microstructure does not compromise culture longevity with regard to nutrient supply, as the entire PDMS structure is immersed in approximately 1 mL of liquid (see Fig. S5† for a schematic representation). Note, the PDMS sticks to the surface solely through adhesion forces.

### 2.3 Cell culture

The medium used for culturing the cells is NeuroBasal medium (NB) (21203-049, ThermoFisher).^55^ NB complete medium was prepared freshly. NB complete medium is 2% solution of B-27 supplement (17504-044), 1% solution of penicillin–streptomycin (P–S) (15070-063) and 1% solution of GlutaMAX (35050-061, all from ThermoFisher).

#### 2.3.1 Cell dissociation

Primary thalamic or cortical neurons from E18 embryos of pregnant Sprague–Dawley rats (EPIC, ETH Phenomics center) were used in the experiments. Animal experiments were approved by the Cantonal Veterinary Office Zurich and performed in accordance with the animal welfare laws of Switzerland (TSchG and TSchV) and policies of ETH Zurich. Embryonic neuronal tissue was dissected and stored in hibernate E medium (ThermoFisher A1247601) on ice. Cell dissociation began by digesting the tissue in a solution consisting of 50 mg mL^−1^ bovine serum albumin (BSA) (A7906, Sigma-Aldrich), 1.8 mg mL^−1^d-glucose (Y0001745, Sigma-Aldrich), and 0.5 mg mL^−1^ papain (P5306, Sigma-Aldrich) dissolved in sterile PBS. Directly prior to dissociation, the solution was warmed to 37 °C, filtered (0.2 μm) and 1 mg mL^−1^ DNAse (D5025, Sigma-Aldrich) was added. Tissue was left in the papain solution for 15 minutes at 37 °C, after which the solution was replaced by NB medium with 10% fetal bovine serum (10500056, ThermoFisher) to stop the digestion. Two subsequent washes with NB were done, waiting 5 minutes between each wash. This was followed by trituration and cell counting (Cell Countess, Invitrogen). Cells dissociated from a single pregnant rat were considered one biological replicate.

#### 2.3.2 Cell seeding

Cells were seeded either as spheroids or in suspension, depending on the experiment.

#### 2.3.3 Spheroid preparation and seeding

In the case of microstructures described in section 2.1.1, cells were seeded as spheroids. To prepare the spheroids, after counting the cells, the volume of cell suspension needed to create 500-cell spheroids was added to an AggreWell microwell plate (Stemcell Technologies Inc., Canada). The wells were then filled with NB to a volume of ∼2 mL. The AggreWell containing the cells was centrifuged for 3 min to ensure homogeneous cell distribution and formation of spheroids. The spheroids in the AggreWell were kept in the incubator until seeding.

The day after dissociation, spheroids formed in the AggreWells were ready for seeding in the PDMS microstructures. For seeding, 0.5 mL of spheroid solution was transferred to a small Petri dish to avoid leaving the whole AggreWell at room temperature and without CO_2_. 5–10 spheroids were aspirated from a Petri dish using a 10 μL pipette and a spheroid was carefully pipetted into the center wells of the microstructures one-by-one. Once all seeding spots were filled but no longer than 10 min after the spheroids were removed from the incubator, the culture (a glass dish or a MEA) was placed in the incubator at 37 °C, 90% humidity and 5% CO_2_. In the experiments with spheroids, the day when spheroids were seeded in the PDMS microstructures was considered day *in vitro* (DIV) zero.

#### 2.3.4 Cell suspension seeding

In the case of 75 μm-high microstructures, larger spheroids were too large for seeding into the wells without falling out due to the smaller PDMS thickness and smaller spheroids were too small for manual seeding. Hence, neurons were seeded in suspension. After counting the cells in the solution upon dissociation, the exact volume to obtain roughly 21 000 cells per mm^2^ was calculated. The cells were seeded with a pipette centered at the top of the PDMS microstructures to increase the probability of cells falling inside the wells. 20 min upon seeding, dishes were inspected under the microscope to assess if enough cells have fallen inside the seeding wells. In case there was not enough neurons in the wells, they were re-suspended by pipetting the medium up and down above the PDMS microstructure. In the experiments with such cell suspensions, day of cell dissociation and seeding was considered DIV zero.

#### 2.3.5 Cell maintenance

Cultures with cells confined inside the PDMS microstructures contained ∼1 mL of medium. This was sufficient to completely cover the PDMS microstructures and allow for passive nutrient diffusion inside the microchannels. Twice a week the cell medium was exchanged from the dish under the laminar flow hood by pipetting out ∼0.5 mL of the old medium from the edge of the dish and adding ∼0.6 mL of the fresh warm medium.

### 2.4 Fluorescent labeling

While in the AggreWell prior to seeding, spheroids were labeled with enhanced green fluorescent protein (eGFP) in the AggreWell plate by adding ∼10 k particles per cell of the adeno-associated viral (AAV) vector (V-DJ/2-hSyn1-chl-EGFP-SV40p(A)) (University of Zurich Viral Vector Facility). Cells to be seeded in suspension were transduced with eGFP AAV and mRuby3 (scAAV- DJ/2-hSyn1-chl-mRuby3-SV40p(A)) AAV. Staining cells with AAVs directly upon cell dissociation before seeding them in microstructures offers several advantages. It enables efficient and continuous tracking of axons from the same culture over multiple days. Furthermore, fixing and staining cells within a PDMS microstructure can be challenging due to limited antibody diffusion in the microchannels.

### 2.5 Image acquisition and analysis

Transduced cultures were imaged using a confocal laser scanning microscope (CLSM) (Fluoview 3000, Olympus). The images were acquired using either 20× (Olympus, UPLFLN20XPH, NA = 0.5) or 30× (Olympus, UPLSAP030XS, NA = 1.05) objective, depending on the experiment. One or two channels were acquired in combination with brightfield with a laser wavelength of 488 nm (for GFP-expressing cells) and/or 561 nm (for mRuby-expressing cells). Acquired images were analysed using Fiji^56^ and custom-made Python scripts. To assess the length of axon growth in the microchannels, images were overexposed and the length of the segmented line drawn on top of the axon was measured. See Fig. S6† for details.

### 2.6 Electrophysiology

6-channel and 60-channel spheroid-seeding PDMS microstructures were designed to fit the electrode layout of glass MEAs that were used to performed the electrophysiology experiments. During recording sessions, neurons cultured on MEAs were taken out of the incubator and placed in the MEA headstage (MEA2100-Systems, Multi Channel Systems) and kept at 5% CO_2_ without humidity control during 10 min recordings. The recordings were taken after two and four weeks in culture and each MEA was also imaged once at the end to assess the axon outgrowth.

The collected data sampled at 25 kHz was first filtered using a butterworth high pass filter with a cutoff frequency of 200 Hz. Spike detection was performed based on negative spike peaks. The baseline noise of the signal was calculated using the median absolute deviation (MAD). MAD provides a robust estimate of the standard deviation by being less affected by outliers in the voltage trace. Unlike directly calculating the standard deviation from the spike trace, using MAD yields more reliable results.^57^ The standard deviation *σ* was defined based on MAD as follows:

1 *σ* = *b*xMAD,

where *b* = 1.4826 is a constant defined in the work by Leys *et al.*^58^ The peaks were considered neural spikes if their amplitude exceeded the baseline noise value 5 times. Additional peaks occurring within 3 ms after the first detected peak were discarded to avoid duplicates.

The conduction speed of forward-propagating spike trains was assessed using spike-triggered time histograms (STTH) (see ESI† Fig. S7). This method, which has been previously used to demonstrate functional connectivity among large groups of neurons,^59^ STTHs display the distribution of spike time latencies between two electrodes by plotting the detected spikes on one electrode relative to a spiking event on another electrode. A peak in the latency distribution that surpasses the baseline level indicates a correlation between the spiking events on the two electrodes. The latency of each significant peak in the STTH is directly linked to the spatial distance between the electrodes. For each electrode, spikes occurring within a 16 ms window following a spiking event were accumulated into bins of 0.05 ms. To calculate the conduction speed, the distance between the electrodes was divided by the lower edge of the bin with the highest spike count:2
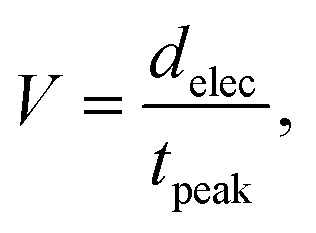
where *d*_elec_ in mm denotes the distance between the two respective electrodes, and *t*_peak_ in ms denotes the lower value of the time bin that contains a peak value of the latency distribution. The upper limit of the time window was chosen way above the expected conduction speed range (the lowest conduction speed would then correspond to ∼0.15 m s^−1^). The example of the conduction speed measurement is depicted on Fig. S7.† More detail about the applied method in the context of patterned *in vitro* neuronal networks can be found in Amos *et al.*^45^ In all electrophysiology data analyses, statistical significance was assessed using the Kruskal-Wallis test.

## 3 Results and discussion

In our investigation of how microstructure design impacts axon growth two similar, but distinct, experimental paradigms were used. For probing the impact of channel count and width we used spheroid cultures to ensure both the formation of axon bundles and to have an excess of axons compared to channels. In experiments investigating growth cone penetration through narrow restrictions the excess of axons was unnecessary and as such, low density cultures and associated microstructures (see section 2.1.2) were used. In all the experiments, we used PDL as a cell-adhesive coating due to the simplicity of application, long term stability, and compatability with PDMS adhesion. Attempts were made to use laminin as a secondary coating, which resulted in poorer PDMS adhesion. Since cell viability was maintained with PDL alone, the laminin coating was removed from the protocol. Additionally, laminin was not added as medium supplement post-seeding to avoid laminin deposition and thus neurite outgrowth on top of the microstructure. The axon growth was compared among identically prepared structures, which avoided adding a bias in the measurements.

### 3.1 Axon growth in spheroid-seeding microstructures with variable microchannel width

We seeded cell spheroids prepared as described in section 2.3.3 consisting of 500 neurons in the central seeding well of the PDMS microstructure. Emerging from the central well, microchannels initially narrow down to 1.5 μm (Fig. 2B) to ensure equal probability of similar number of axons entering each microchannel and then expand to the final microchannel width which increased logarithmically from 1.5 to 75 μm (Fig. 2A). We chose these widths because we believed that differences would be more prominent for lower channel widths (below 10 μm). As seeding large neural spheroids provided an excess of axons, most channels were filled with axons (see S8† for confirmation).

Growth of GFP-labeled axons was assessed by imaging at three time points across three weeks *in vitro*. We imaged the samples at DIV 7, 12 and 23 and measured the extent of the axon length starting from the end of the narrowing/beginning of the final channel (see also Fig. S8†). Since, we uniformly coated the dishes with PDL prior to mounting the microstructures, we expect equal density of axon guiding cues on the bottom of all microchannels, which implies that all putative differences arise from the differences in width of the PDMS microchannels. We observe a steep increase of axon length for microchannel widths up to 2.8 μm (Fig. 2C, D) after which there is a plateau at around 2 mm in length with a high variability at DIV7 (see also Fig. S9†). A similar plateau is observed at 3 mm in length at DIV12 and 23. The growth of axons in topological constraint *in vitro* in two dimensional stiff substrate presents a significant difference when compared with axon lengths measured *in vivo*. Namely, though the variability is high, thalamocortical axon fibers in living rats reach total length of more than 6 mm (ref. 11) which raises questions on the need for extracellular matrix that would provide three dimensional axon projections *in vitro* and thus increase the physological relevance of the *in vitro* platforms.

Notably, axons grow fastest in the first week of culture where projections in microchannels wider than 2.8 μm reach roughly 2 mm in length. Between DIV 7 and DIV 12 the length revolves around 3 mm and it remains at this value at DIV 23. From this we conclude the majority of axon growth happens within the first two weeks in culture, which is consistent with previous findings in the context of synapse formation,^60^ neural activity,^61^ and neurite growth.^62^ Furthermore, it is worth discussing the profile of axons growing in microchannels. On Fig. 2C we notice that axons in all channels tend to grow along the edge of the PDMS wall, which has already been reported elsewhere.^46^ Channels smaller than 11.6 μm in width tend to contain bundled axons, while in the wider channels the bundles tend to split into two sub-bundles each following one of the PDMS side walls (see Fig. 2C).

### 3.2 The number of bundles per microstructure affects axon growth and AP propagation

In the next set of experiments, we report culturing 500-neuron spheroids in spheroid-seeding PDMS microstructures with constant channel width of 50 μm (Fig. 3A). We vary the number of channels that emerge from the central well in order to influence the average size of axon bundle formed in the microchannels. Using 500-neuron spheroids would theoretically limit the number of axons to less than 9 per channel in the 60-channel microstructure, but we do not expect all neurons to project axons outside the spheroids. We expect the largest axon bundles in the 6-channel microstructure, while the 60-channel microstructure should contain the fewest axons per microchannel, hence forming the smallest axon bundles on average. The support of this claim can be found in Fig. S10;† however, we are unaware of quantitative fluorescence methods that allow for accurately counting the exact number of axons in each channel this is a relative measure. Cells were cultured in PDMS on glass substrates and imaged on DIV 23. In Fig. 3B we observe a decreasing trend in length for the increasing microchannel number. Since we assume microstructures with fewer channels emerging from the central well would contain more axons per channel, we expect thicker axon bundles in these microstructures than the axon bundles forming in, *e.g.* 60-channel microstructures. Hence, we conclude that thicker axon bundles tend to grow further. This finding might be related to the role of the aforementioned axon-axon interaction in axon growth,^53,63^ namely that there is a higher probability of finding a pioneer axon that would direct the growth in larger axon bundles.

Another possible explanation is that in 60-channel microstructures, in which the few axons can explore the full 50 μm width of the channel, the increased available space may lead to axons changing direction more often during growth instead of growing straight along the microchannel (and adjacent axons). This conjecture regarding the influence of spatial constraints can be further substantiated by analyzing the morphology of axon bundles within these microchannels. Specifically, in the case of 6-channel microstructures designed to accommodate larger axon bundles, axons uniformly occupy all available space, likely facilitated by the higher density of axons within the bundle (Fig. 3C). Conversely, in 60-channel microstructures, axon growth patterns resemble those observed in the experiments introduced in subsection 3.1. Namely, they tend to form sub-bundles and follow the edges of the surrounding PDMS walls. We also believe that punctuated axon morphology is actually axon varicosities,^64^ which are more pronounced in cultures with fewer axons. This increased prominence likely occurs because, in these conditions, axons tend to spread out across the 2D substrate rather than forming dense bundles, which makes the varicosities more visible. Lastly, similar to the case of the microstructure with different channel widths, we observe high variability of the axon length as implied by the broad Gaussian distribution, especially in the case of the 6-channel microstructures.

Next, we placed the microstructures with the smallest (6) and the largest (60) number of microchannels on top of the MEAs to study the electrophysiological properties of axon bundles consisting of different number of axons. We calculated the mean firing rate per channel and the AP conduction speed as described in section 6. We aligned 6-channel and 60-channel microstructures on top of MEAs using a brightfield microscope to obtain as many electrodes as possible per microchannel. This offered maximum 12 channel replicates per MEA. For a 60-electrode MEA of array shape 6 × 10 used in this experiments, the maximal number of electrodes per channel was five. The two electrophysiology metrics presented here *i.e.*, firing rate and AP conduction speed, serve to give an idea of the viability, general level of neural activity, and successful AP propagation within these devices. We assume other electrophysiology metrics assessing the excitability, or related metrics, of the network^65^ will be more heavily dependent on the underlying cell types and connectivity in each node rather than on the number of outgoing connections, while metrics regarding the direct information content within a channel, even for patterned networks, are typically relevant only in stimulation experiments,^45,57^ though this is liable to change as the complexity of engineered networks grows.

In the case of 60-channel microstructure shown in Fig. 4A, due to the limitation on the number of electrodes, only a selection of channels could be recorded from, whereas the 6-channel microstructure (Fig. 4B) was aligned on top of a MEA to obtain the recording from all 6 available channels. While some electrodes experienced noise levels as high as 0.2 mV (peak-to-peak), many action potentials exhibited amplitudes of 0.4 mV or greater (see Fig. S11†). The amplification of a signal is a consequence of insulation provided by the PDMS microchannel. This significant difference made the action potentials easily detectable by our spike detection algorithm. The MFR was calculated per channel, thus providing a measure of an activity of axon bundle within a channel, rather than a single cell within a bundle. The extraction of single cell activity is in this case difficult also due to the potential variability of the spike shape across the same axon, which has been shown earlier for extracellular electrophysiology data in the microchannels.^66^

In Fig. 4C we observe an increase of MFR per channel from week 2 to week 4 in culture. In fourth week in culture, we note a significant difference between MFRs for neurons in 6- and 60-channel microstructure respectively. In 60-channel microstructures, where there is presumably a lower density of axons per channel, we observe a higher MFR. This phenomenon can be attributed to the reduced number of axons within the microchannel, potentially enabling some of them to attain larger diameters within the same microchannel cross-section. Consequently, APs of these axons generate larger extracellular potentials, rendering them more readily detectable by the electrode. There is another hypothesis stemming from our findings if we consider previously stated assumption that a lower number of microchannels could results in a higher density of axons per microchannel. This denser arrangement might limit the diffusion through the channels by increasing the tortuosity of the channels, thereby slowing down glucose and nutrient exchange and potentially leading to reduced neuronal activity.^48^

In Fig. 4D we calculated AP conduction speed for axons confined in 6- and 60-channel microstructure respectively for 4-week old cultures. Although there is no significant difference between them, we observe that the mean conduction speed for axon bundles within a 60-channel microstructure is lower, though the variability is higher. The higher variability in conduction speed for 60-channel microstructures can also be attributed to potentially having less axons per microchannel. Namely, as stated before, the excess of space allows for some axons to grow larger in diameter. Since the conduction speed depends also on axon diameter, higher variability in diameter could cause higher variability in conduction speed.

### 3.3 Growth cones able to penetrate 600 nm wide tunnels

In our subsequent experiments, we further explored the spatial confinement of axons using PDMS microchannels. Our aim was to probe the boundaries of axon growth and the flexibility of growth cones. Specifically, we sought to determine the minimal width of confinement required for a growth cone to adjust its size and traverse through. In order to eliminate the influence of other axons, we opted to seed cells in low-density suspensions.

**Fig. 5:**
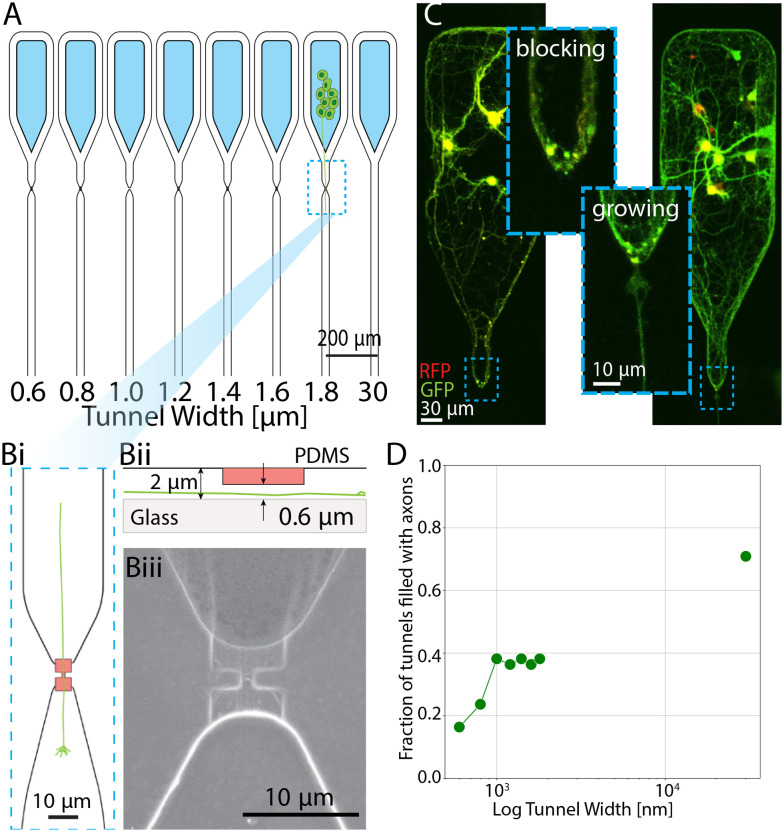
PDMS microstructures for studying axon growth through confinement. A) Neurons seeded into the seeding wells (blue) extend their axons towards the submicron-sized tunnel guided by narrowing microstructures. The tunnel width varies from 600 to 1800 nm while the subsequent microchannels have a fixed 30 μm width. Bi) Schematic of the submicron tunnel region. Ideally axons grow directly through the narrowing. Bii) The tunnels are 600 nm high, while the rest of the channel has a height of 2 μm. Biii) SEM image of submicron narrowing. C) Examples of axons turning and blocking the tunnel on the left and passing through the tunnel on the right. D) Fraction of tunnels that have axons passing through. There are 6 biological replicates.

In the first set of experiments, we designed a microstructure that consists of eight separate seeding wells that narrow down to a 30 μm wide and 2 μm high microchannel to ensure the axon guidance towards the microchannel (see Fig. 5A). At the beginning of each microchannel there is an additional PDMS layer that narrows the available vertical space for axon growth to 600 nm (see Fig. 5B). This narrowing we refer to as a submicron tunnel. Tunnels vary from 0.6 to 1.8 μm in width presenting a spatial limitation in both horizontal and vertical direction. The last, eighth well does not begin with a tunnel but serves as a control channel of the axon growth in general. In the case of cell-suspension seeding microstructures, we specifically looked at yes/no events *i.e.* whether the growth cone would penetrate the submicron tunnel or not. Typically, this occurred within the first two weeks in culture, after which the submicron tunnel area became densely packed with cell material (as shown in Fig. 5C). These assumptions are further backed by the fact that axons perform majority of exploration within the first couple of days after seeding (see ESI† videos) and the fact that synapse maturation occurs in the first part of the second week in culture.^60^ Therefore, we believe that, while it is possible that axons would have still been able to navigate to the tunnels, it is more likely that it has already followed another path. The data presented in Fig. 5 is for the last imaging point of the experiment (DIV 12).

Instances depicted in Fig. 5C on the left, where axons curved and obstructed tunnel entrances, were noted. On the right of Fig. 5C, we can observe an instance of an axon bundle narrowing down to successfully pass the tunnel. We observed that tunnels with widths equal to or larger than 1 μm reached a plateau, with approximately 40% of these tunnels being filled (see Fig. 5D). Similar trends were found in the experiments performed with cortical and thalamic neurons respectively (see Fig. S12†), though the data suggests that cortical neurons more frequently fit through narrow tunnels. Our findings suggest that even with openings up to 1.8 × 0.6 μm^2^, neurons did not exhibit consistent growth. The impaired growth potential is an important aspect to be considered when designing microfluidic devices or an experimental setup in neural applications since it can affect the development of the cultured cells. If consistent growth is desired, both the narrowest point of the design and the channel width are important factors, as indicated by the results presented in this section and subsection 3.1.

In the context of growth cone flexibility and the constraints on axon growth, we found that a width of 600 × 600 nm^2^ still allows axons to penetrate through in about 18% of our experiments. Considering that the average axon diameter is approximately 450 nm,^67,68^ and acknowledging the flexibility and adaptability of growth cones to their environment, this outcome is consistent with expectations.

### 3.4 Growth cones cannot penetrate tunnels narrower than 350 nm

**Fig. 6:**
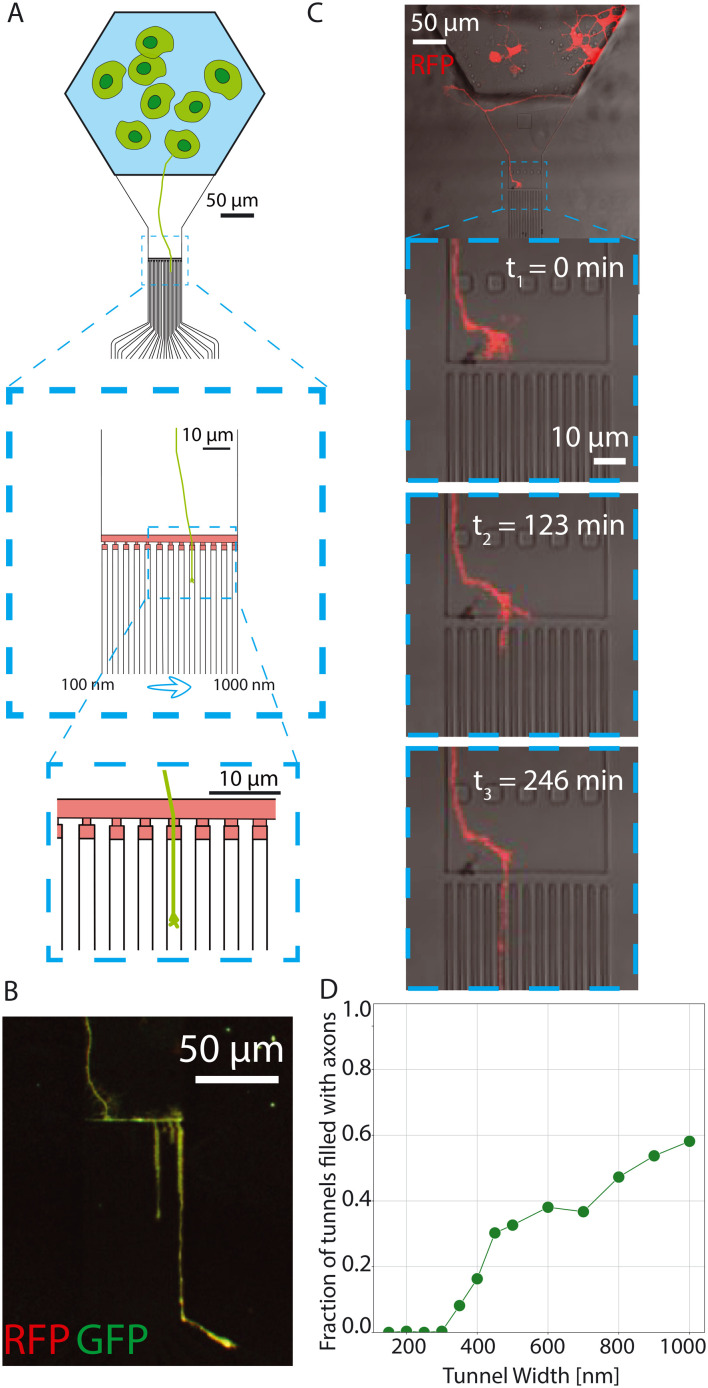
PDMS microstructures with smaller tunnels for studying the spatial limitations of axon growth. A) Microstructure schematic. Cells seeded in suspension fall into a hexagonal well. The axons are guided towards a submicron tunnel area and penetrate through. B) Example of a single axon branching through multiple tunnels. C) Overnight time lapse at DIV 2 (ESI† Movie S1). D) Fraction of tunnels filled with channels. There are 6 biological replicates.

Since in the previous section we have shown that axons can transverse through constrictions as small as 600 nm, in the last set of experiments, we decided to explore the very limitations of axon growth. We used the microstructure consisting of hexagonal seeding wells that narrow down to a 50 μm wide and 2 μm high area leading to a series of submicron tunnels that are 600 nm high (depicted in red in the zoomed in region of Fig. 6A). With this microstructure we investigated tunnel widths varying from 100 to 1000 nm. In the previous set of experiments we observed that excess cell material at the narrow crossing points often blocked the tunnel entrance, so we designed this microstructure such that multiple tunnels share the same, wider microchannel. The open node transitions to 2 μm high area that narrows down to a 600 nm area, which contains the submicron tunnel. Hence, each tunnel is 600 nm high but varies in width in the aforementioned range (100 to 1000 nm).

To investigate if growth cones are able to penetrate the tunnels, we imaged the cultures in the second week in culture at DIV 10. We also performed an overnight time lapse on a subset of cultures on DIV 2 and DIV 3 to track axon movement and growth cone flexibility (full video is available in the supp. material). In Fig. 6B we observe a putative single axon growing along the tunnel area and splitting to penetrate three tunnels (for full size image see Fig. S13†). The snapshots of the overnight time lapse video shown in Fig. 6C were taken at *t*_1_ = 0 min, *t*_2_ = 123 min and *t*_3_ = 246 min. We observe the growth cone of approximately 8 μm in diameter conforming its shape to fit into the 500 nm wide and 600 nm high tunnel. We notice that the same amount of time was needed for the growth cone to penetrate the tunnel and for the axon to afterwards grow for approximately 50 μm. This is consistent with previous research that implies an increase of growth velocity in a topological constraint *versus* planar surfaces.^69^

We did not observe any axons penetrating tunnels smaller than 350 nm. For wider channels we observe a gradual increase with channel width to reach the value 50% of channels with axons at 1 μm, which is slightly higher than what we observed in the experiments described in Fig. 5D. This could be due to the aforementioned smaller likelihood of channels getting blocked by cell material with this design. This observation indicates that axons are more likely to enter wider microchannels. Similar overview of the effect of topographical constraints on axon growth has been shown.^49^ These results further highlight the importance of micro−/submicro-environmental factors on axon growth.

## 4 Conclusions

We presented a comprehensive study focusing on axon growth within a spatially constrained environment for more than three weeks *in vitro*, with axon length changing minimally after DIV 14. We showed that while geometric restrictions can reliably limit the number of axons growing through a channel, down to single axons when the critical dimension of the restriction is between 350 nm and 450 nm for the neuron types tested, this limits the overall length axons grow on average if the restriction persists across a substantial length. However, even though limitation of axon number is reliable, it is conditioned upon the likelihood of a growth cone passing through said restriction. This is heavily dependent on the agglomeration of the cell material in the proximity of the restriction, *i.e.*. The agglomeration of cell material can to some extent be prevented by lowering cell seeding density, thus enabling low amount of cells per seeding well. Increases in the channel width after restriction beyond 3 μm show axons have a median length of 3 mm ± 1 mm which is similar to the average axon length when there are 36 or more unrestricted efferent channels, indicating the transient restrictions of 1.5 μm are unlikely to be the primary factor limiting axon growth. While larger axon bundles grow on average 1.5 mm longer, we are able to record a higher firing rate in channels with fewer axons. Whether this is due to a smaller portion of the axons being in the sensing volume of the electrode or due to a true difference in activity requires further investigation as the latter would imply a greater role of the axon in signaling than a transmission cable.^70^ Nevertheless, there appears to be a tradeoff between the sparsity of axons, thereby the granularity of the guidance, and the length axons are able to grow. Further technical advances, such as incorporating within the channels either hydrogels functionalized with axonotrophic molecules or support cells, *e.g.* Schwann cells, may mitigate this and should be explored further.

Though we were able to determine the minimum size axons would be able to pass, open questions remain regarding whether there were physiological differences between those neurons crossing at different apertures. Nevertheless, we hypothesize that for multiple axons to traverse a submicron channel that the cross section would need to be at least 700 × 600 nm meaning single axon guidance can be achieved with ∼40% yield, with a higher yield possible when it would be acceptable for there to be a chance of two axons crossing. This could overcome one challenge faced in brain-on-a-chip applications, patterning pre- and post-synaptic partners, which would allow for a broad range of experiments investigating the functional role of an individual neuron or axon, *e.g.* on synaptic integration, or controlling the total number of afferent axons between two neural populations. Additionally, studies investigating both axonal and growth cone physiology of individual axons can be made, for example long term examination of cytoskeletal structure during growth or in response to stimulation.

While this study was limited to cortical and thalamic neurons, since the cortico-thalamic pathway is an attractive target for testing biohybrid devices in animal models,^71–73^ the methodology used here can be tailored to other neuron types. The growing repertoire of cell type specific human induced pluripotent step-cell derived (hiPSC) neurons from individual patients is already increasing the translational impact of brain-on-a-chip devices; however, as neural morphology differs across brain regions and more so across species the dimensions for each device will need to be modified accordingly.

Additionally, we have identified some areas for potential improvement for similar experiments. Some microchannels in this work extend up to 8 mm, which to an extent limits nutrient exchange for axons within the microchannels even though they are open at the distal end. This challenge can be tackled by making the channels fully open^74^ or partially open (*e.g.* introducing diffusion wells along the channels) to facilitate nutrient diffusion. Employing techniques, for instance atomic force microscopy combined with fluidics,^75^ would afford greater control over the size of neural networks and axon bundles forming in the microchannels. Using either iPSC lines or cell sorting prior to cell seeding would allow investigating the difference in growth between specific neural subgroups with greater precision than using tissue from particular brain regions as was done here. Furthermore, in this work we have investigated axon dynamics of rat neurons dissociated at embryonic day 18. It can be assumed that neuronal morphology would vary across different developmental stages, thus the methodology would again need to be adjusted accordingly.

In summary, this work has shown how design decisions about channel geometry influence axon growth ranging from a handful of large efferent bundles down to guiding single axons. We anticipate these findings can be used to further probe single axon dynamics or in guiding the design of neurofluidic devices.

## Data availability

Data is available from the ETH Research Collection at doi: https://doi.org/10.3929/ethz-b-000691498.

## Author contributions

KV: conceptualization, methodology, software, validation, formal analysis, investigation, writing, visualization. GA: conceptualization, methodology, software, investigation. TR: conceptualization, methodology, validation, investigation, data curation, review & editing, supervision, project administration, funding acquisition. RK: investigation. SI: conceptualization, methodology, software. JK: software. JV: conceptualization, review & editing, resources, supervision, project administration, funding acquisition. SW: conceptualization, methodology, software, validation, investigation, review & editing, visualization, supervision, project administration.

## Conflicts of interest

There are no conflicts to declare.

## Supplementary Material

LC-024-D4LC00440J-s001

LC-024-D4LC00440J-s002

LC-024-D4LC00440J-s003

LC-024-D4LC00440J-s004
