# Supplementary material for: Impact of microchannel width on axons for brain-on-chip applications
Source: Lab Chip. 2024 Oct 23;24(22):5155–66. doi: 10.1039/d4lc00440j (PMC11497309; doi:10.1039/d4lc00440j)
Supplement: LC-024-D4LC00440J-s001 [file LC-024-D4LC00440J-s001.pdf]

Supplementary Information (SI) for Lab on a Chip.
This journal is © The Royal Society of Chemistry 2024

Supplementary material

In the Supplementary Table 1, one can find the list of all the PDMS microchannel and (sub)micron tunnel widths used in the experiments with variable channel width, *i.e.* experiments described in Fig. 3, 5, and 6. For the experiments described in Fig. 3 and 4, microchannel size was constant, 50 μm with a microchannel height of 4 μm.

In the Supplementary Table 2, one can find the list of values obtained when performing a Kruskal-Wallis test for assessing the differences in the electrophysiology data.

The global layout of all microstructures can be found in the following figures: spheroid-seeding microstructure used for studying axon growth dependence on channel width (Fig. S1), spheroid-seeding microstructures used for studying axon growth and electrophysiology dependence on channel number (Fig. S2), cell-suspension seeding microstructures used for exploring the limitations of axon growth (Fig. S3 and Fig. S4). All of these designs are freely available upon request from the authors or from Wunderlichips.

Principle of two ways of cell seeding is shown in Fig. S5.

An example of an STTH is shown in Fig. S7 for three channels. The peak of the histogram is detected for every electrode and the distance between the corresponding electrode and the trigger electrode is divided by the time value of the lower edge of the peak time bin to get the conduction speed.

On Fig. S8B) we can observe a pixel intensity profile along the arc that intersects the initial 1.5 μm narrowing of the microchannels, as shown in Fig. S8A). The profile shows uniform distribution across all microchannels (Fig. S8C)), implying the equal abundance of axons per channels. With this assumption, we can attribute all differences in the extent of axonal growth to topological constraints introduced by varying microchannel width.

To assess the extent of axon growth in the microchannels, image was log-processed in Fiji is ImageJ software for better visibility of fluorescence inside the channels. The same software was used to manually draw a segmented line along the channel until the fluorescence was visible by eye, as shown in Fig. S6. The length of this segmented line was used for further calculations.

In Fig. S9 we show the mean length and the coefficient of variation for microchannels with variable channel width described in Fig. 2. Here we want to emphasize higher variability in length for smaller microchannels and additionally higher variability for earlier stages in culture.

An example of superposed action potentials (APs) recorded on the electrodes inside a channel is shown in Fig. S11. APs vary in shape and amplitude.

All microstructure types with GFP-expressing neural spheroids used in experiments described in Fig.3 and 4 are shown in Fig. S10A) and Fig. S10C). All microstructures in the same row are seeded on the same dish and were imaged together, hence enabling the comparison of pixel intensity inside the channels. Average pixel intensity of a part of a channel was calculated for each microstructure. On Fig. S10B) and D) we see a decrease in intensity, implying the lower number of axons per channel for microstructures with higher number of channels.

Fig. S13 is the zoom out of the Fig.6 B), showing a neurite outgrowth inside the seeding well and branching of the axons in the submicron tunnels.

The comparison of the penetration probability trends for cortical and thalamic growth cones is shown in Fig. S12. The trends look similar for both cell types.

Examples of putative single axons and/or axonal branches penetrating micron nad submicron tunnels from

a neurite bundle that formed at the interface. This images were taken at DIV 12 (Fig. S14A))and DIV 10 (Fig. S14B)) and are showing that the tunnels serve as filters.

Table 1: List of channel and tunnel sizes for microstructures with variable width.

Spheroid-seeding microstructure channel size		Cell-suspension-seeding seeding microstructure channel size		Cell-suspension-seeding seeding microstructure channel size	
Width [µm]	Height [µm]	Width [µm]	Height [µm]	Width [µm]	Height [µm]
1.5	4	0.6	0.6	0.15	0.6
1.8	4	0.8	0.6	0.20	0.6
2.2	4	1.0	0.6	0.25	0.6
2.6	4	1.2	0.6	0.30	0.6
2.8	4	1.4	0.6	0.35	0.6
3.2	4	1.6	0.6	0.40	0.6
4.6	4	1.8	0.6	0.45	0.6
5.5	4	30.0	0.6	0.50	0.6
6.7	4			0.60	0.6
8	4			0.70	0.6
9.7	4			0.80	0.6
11.6	4			0.90	0.6
14	4			1.00	0.6
16.9	4				
20.4	4				
24.5	4				
29.5	4				
35.6	4				
42.9	4				
51.7	4				
62.3	4				
75	4				

Table 2: Kruskal-Wallis Test for the Mean Firing Rate and Conduction Speed

Mean Firing Rate (6 vs. 60 channels)			Conduction Speed (6 vs. 60 channels)		
DIV	p-value	Statistic	DIV	p-value	Statistic
12-16	0.02608	4.95086	30-37	0.44969	0.57142
30-37	0.07414	3.18876			

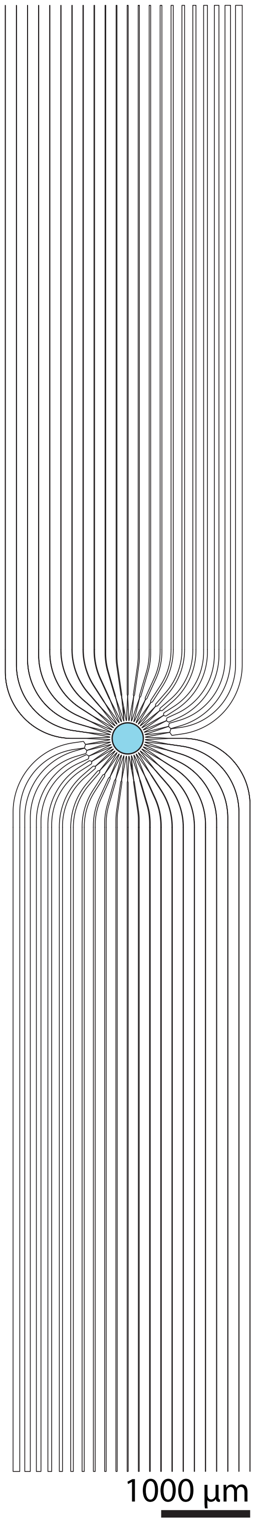

Figure S1: Global layout of the microstructure shown in Main Figure 2 for studying axon growth dependence on the channel width. The spheroid is seeded in the central well and the axons are allowed to expand in the microchannels which are up to 8 mm long.

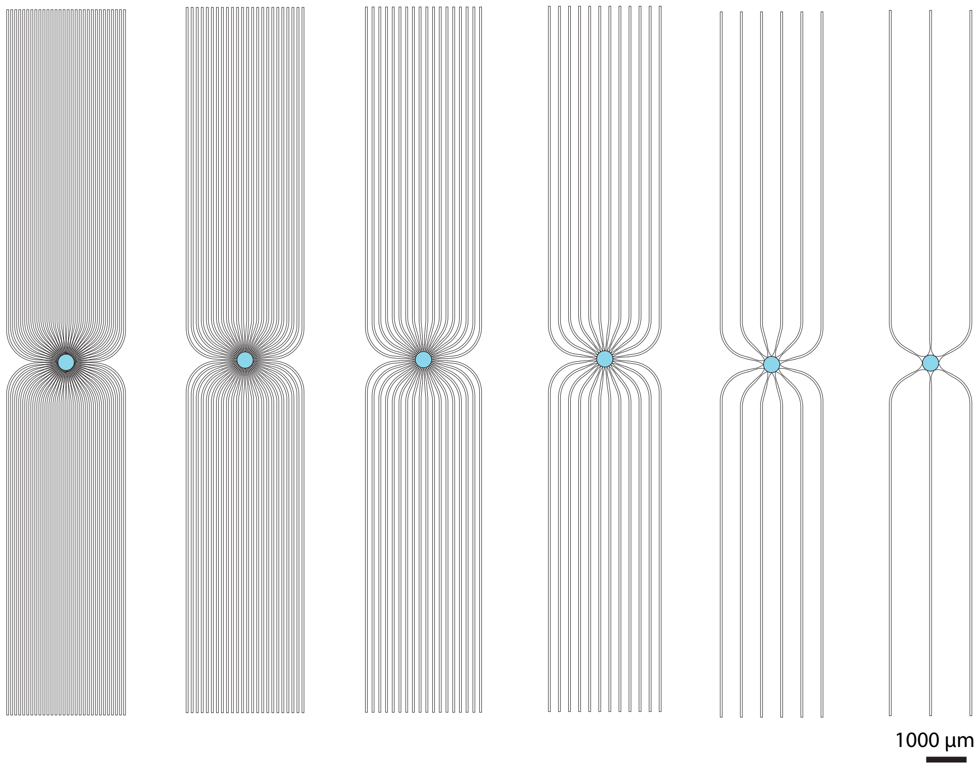

Figure S2: Global layout of the microstructure shown in Main Figures 3 and 4 for studying axon growth and electrophysiology dependence on the number of microchannels (*i.e.*, the available growth sites). The spheroid is seeded in the central well and the axons are allowed to expand in the microchannels which are up to 8 mm long.

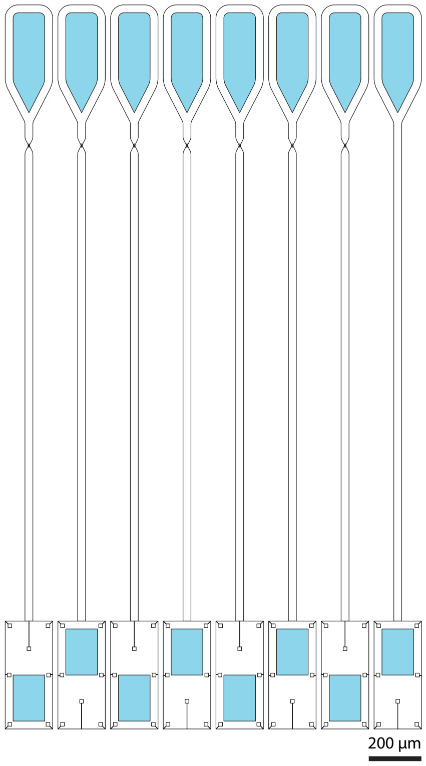

Figure S3: Global layout of the microstructure shown in Main Figure 5 for exploring the limitations of axon growth. Cells in suspension are seeded and axon penetration is observed at the submicron tunnels located at the narrowing point of the funnel-shaped well.

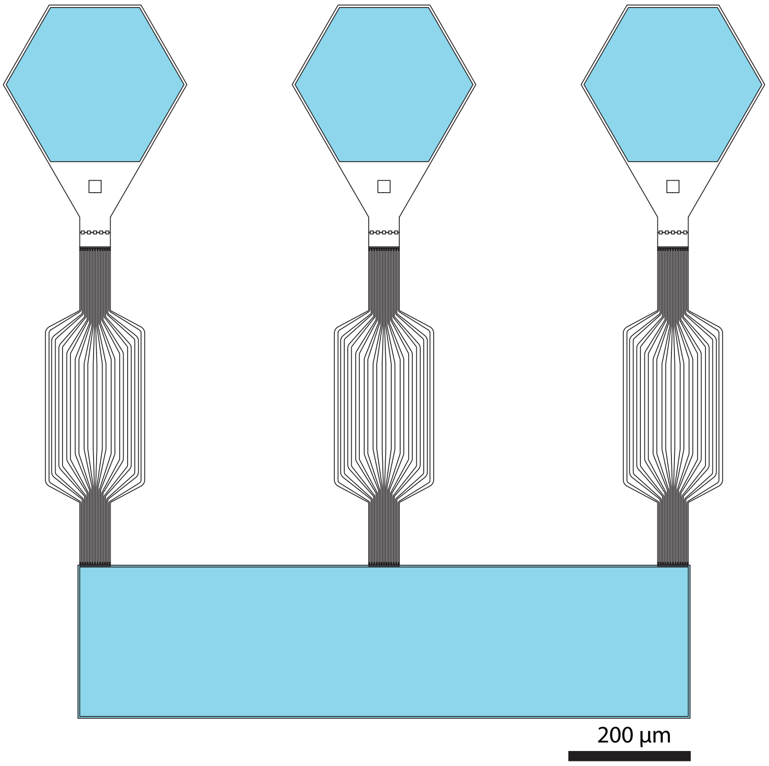

Figure S4: Global layout of the microstructure shown in Main Figure 6 showing three replicates. Cells in suspension are seeded and axon penetration is observed at the submicron tunnels at the narrowing point of the seeding well.

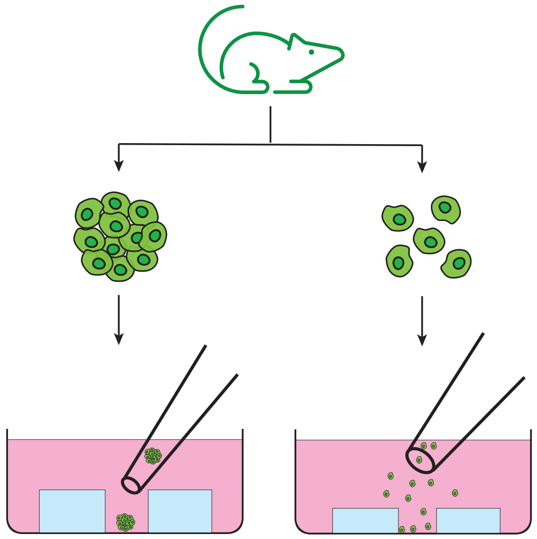

Figure S5: Two methods of seeding cells. Left: Spheroid seeding. 500-cell spheroids are pushed directly into the PDMS well using a 10 μm pipette. Right: Pipetting cell suspension above the PDMS using a 100 μm diameter glass pipette. Medium level is depicted in pink.

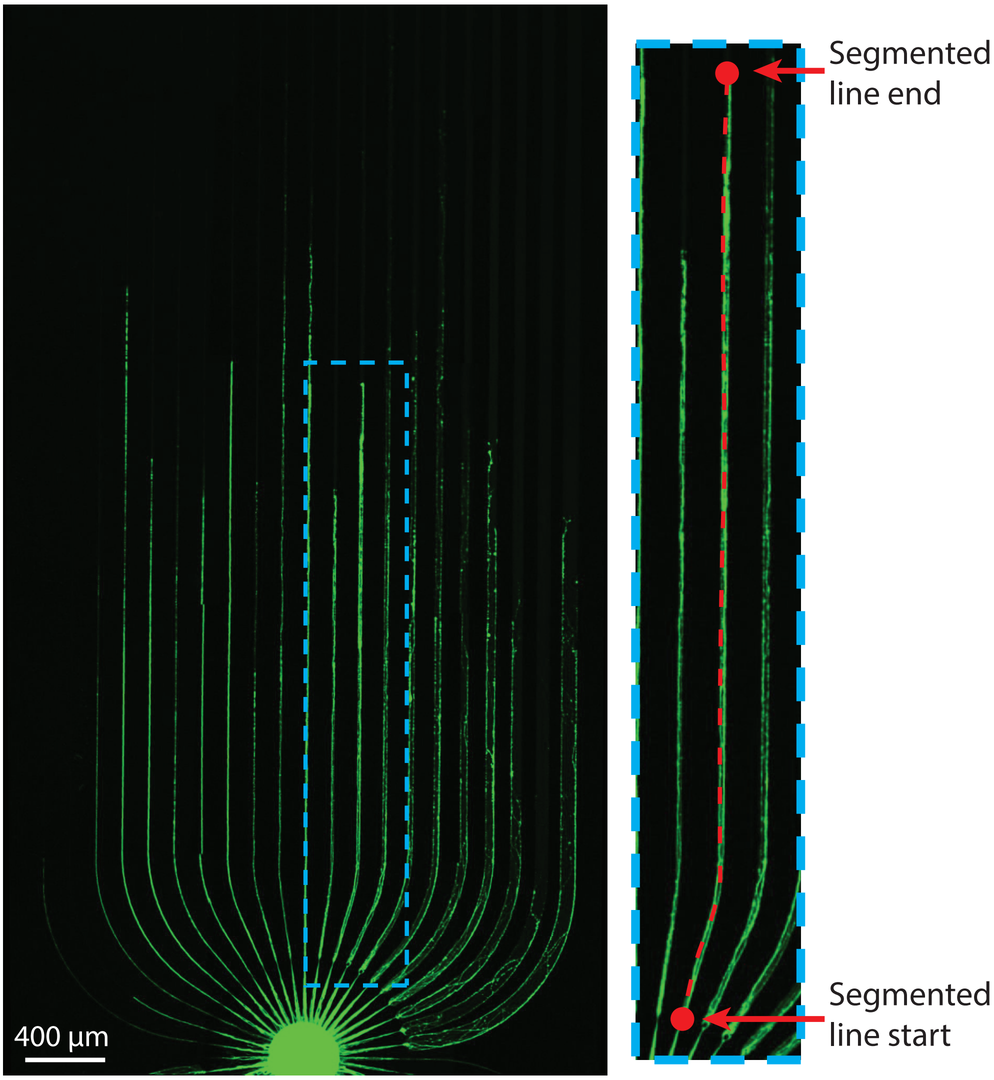

Figure S6: Analysis of the extent of the axon growth in spheroid seeding-microstructures. A segmented line was drawn and the length of the line was calculated using Fiji in ImageJ. The line begins at the end of the 1.5 μm microchannel and ends where there is no fluorescence anymore.

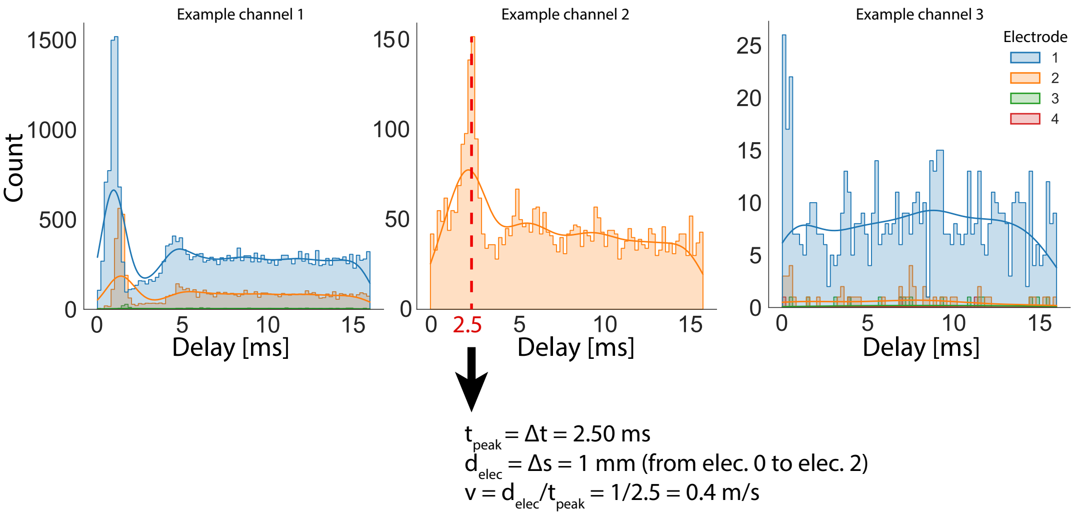

Figure S7: Spike time triggered histogram examples with the first electrode in the channel used as a trigger.

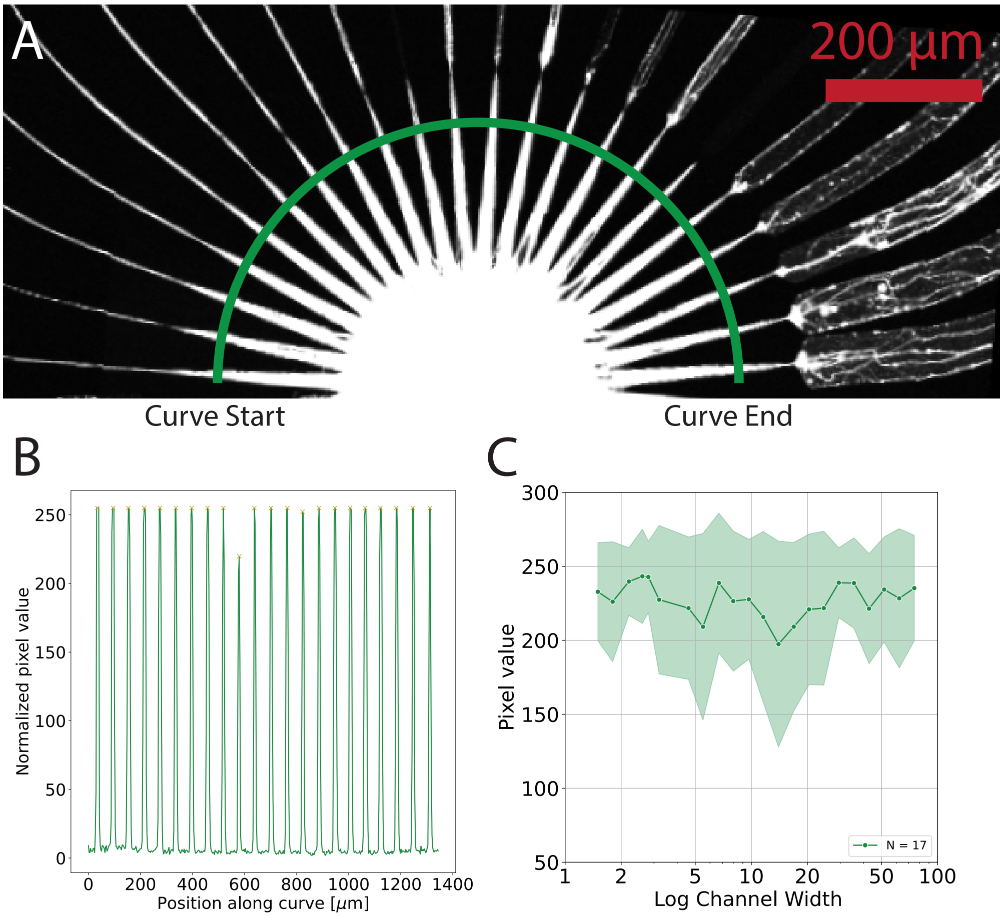

Figure S8: Spheroid-seeding microstructures with variable channel width. A) All channels are initially narrowed down to a 1.5μm wide channel to ensure equal distribution of axons. B) Intensity profile of a semi-circle intersecting the 1.5 μm microchannels. C) Average peak intensity per channel for 17 random samples.

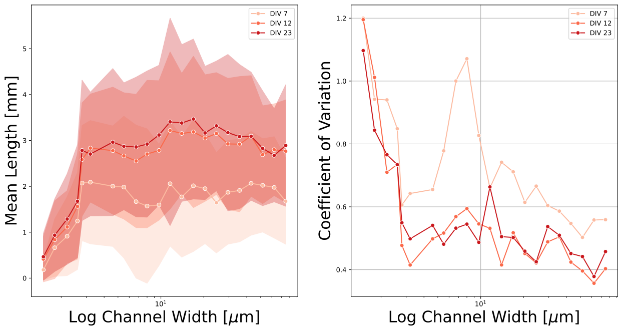

Figure S9: Mean and standard deviation of axon length in spheroid-seeding microstructures with variable microchannel width for different DIV.

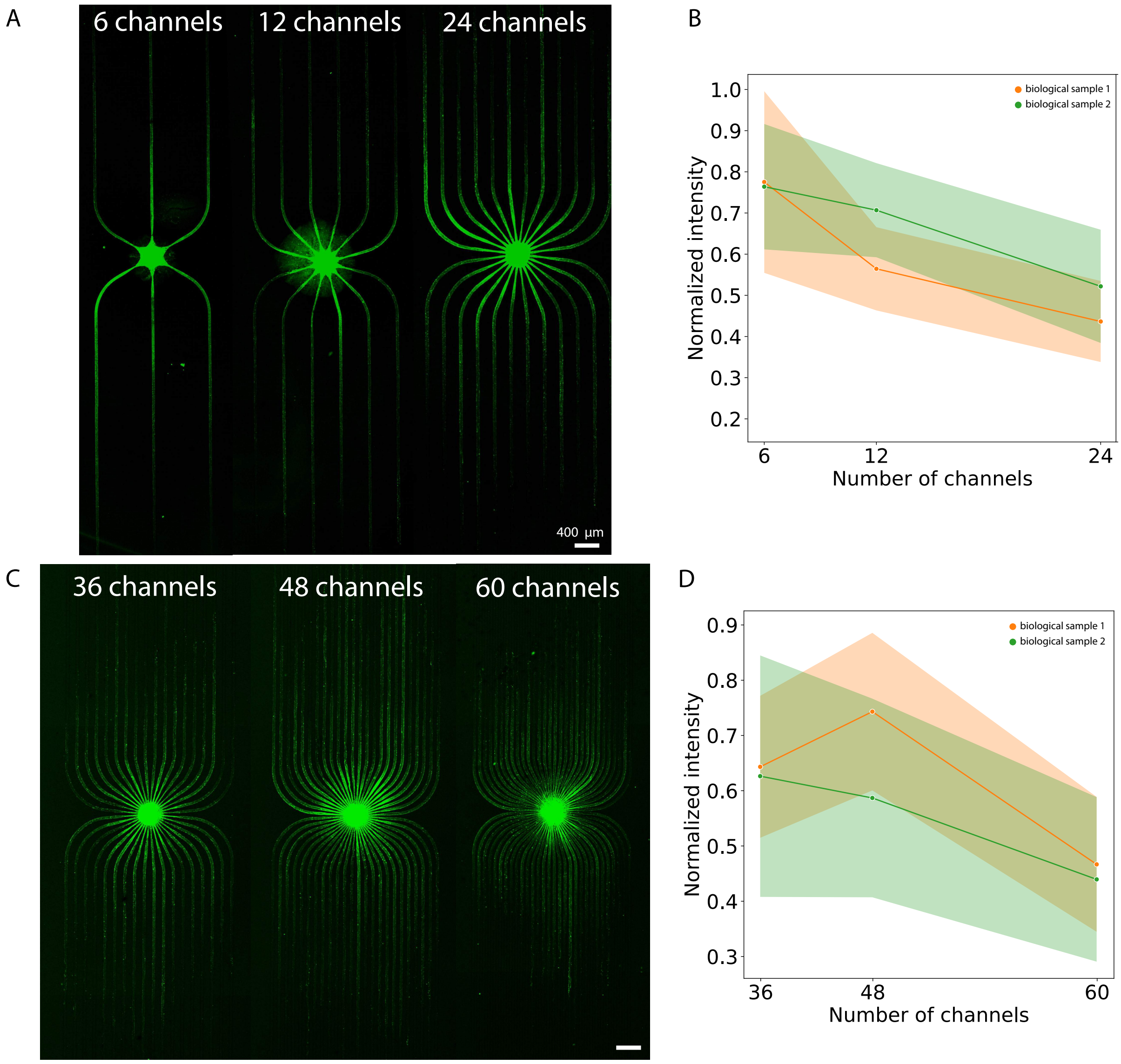

Figure S10: Examples of axon growth in spheroid-seeding microstructures with variable channel number. The pixel intensity within the channel was used as a metric for comparing the bundle size. We observe a decrease in mean pixel intensity for increasing number of channels, indicating a smaller bundle size when there are more microchannels emerging from the central well.

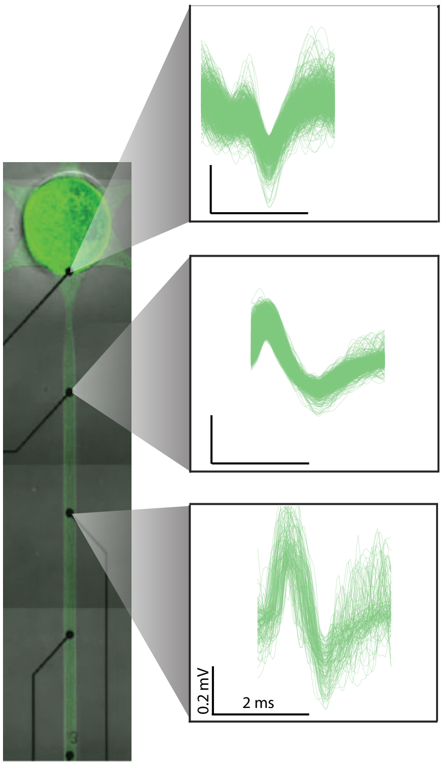

Figure S11: Example of superposed action potentials recorded along three consecutive electrodes in a channel.

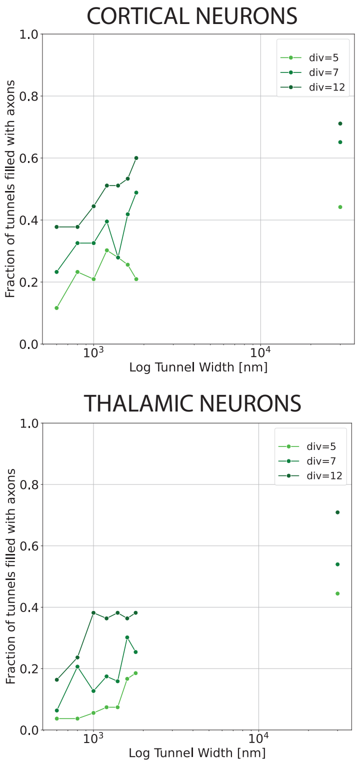

Figure S12: Probability of growth cone penetration into submicron tunnels in the range 0.6-1.8 μm for the microstructure shown in Main Figure 5 for cortical and thalamic cells.

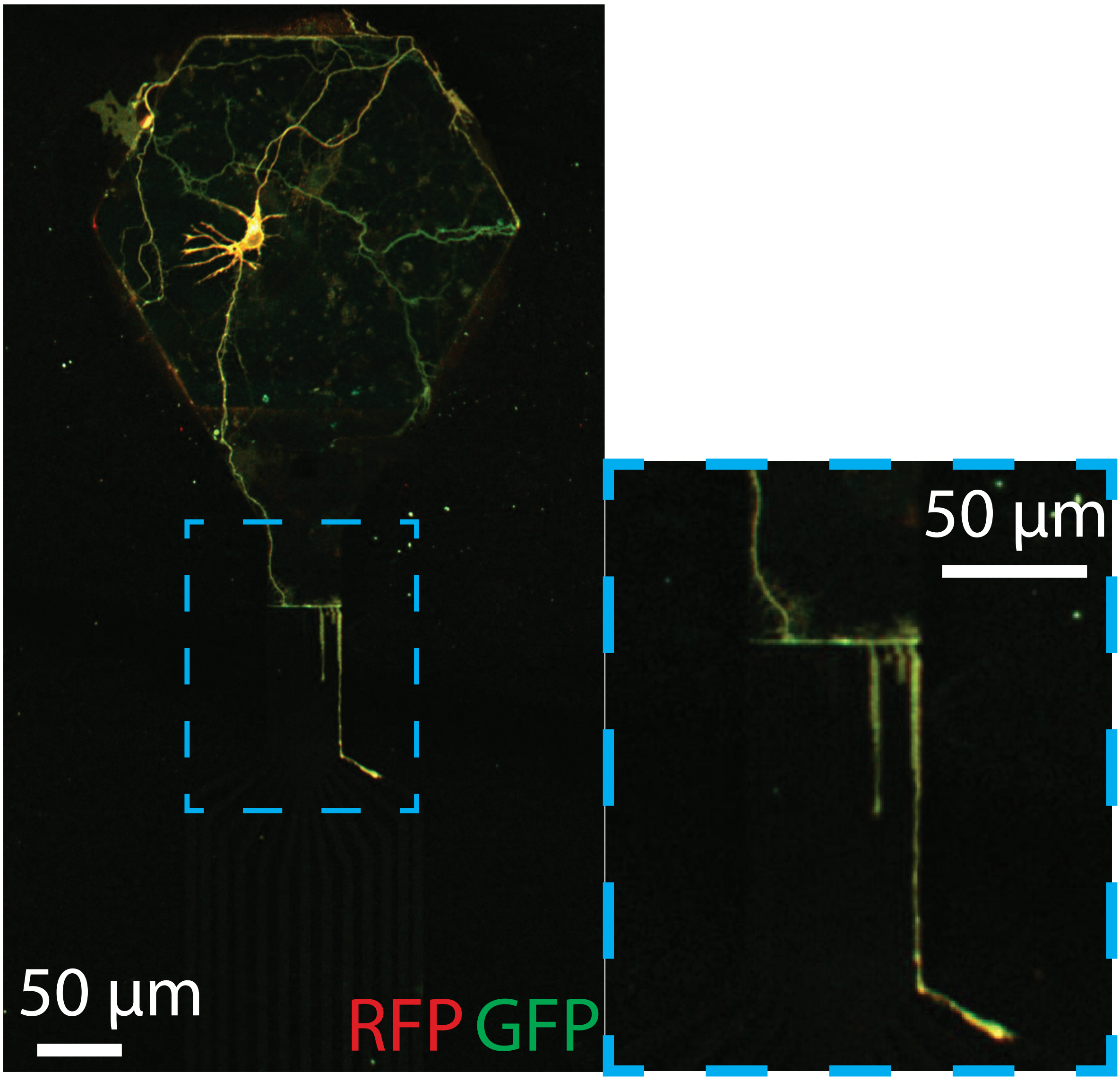

Figure S13: Overview of axon branching observed in the submicron tunnels shown in Main Figure 6 B).

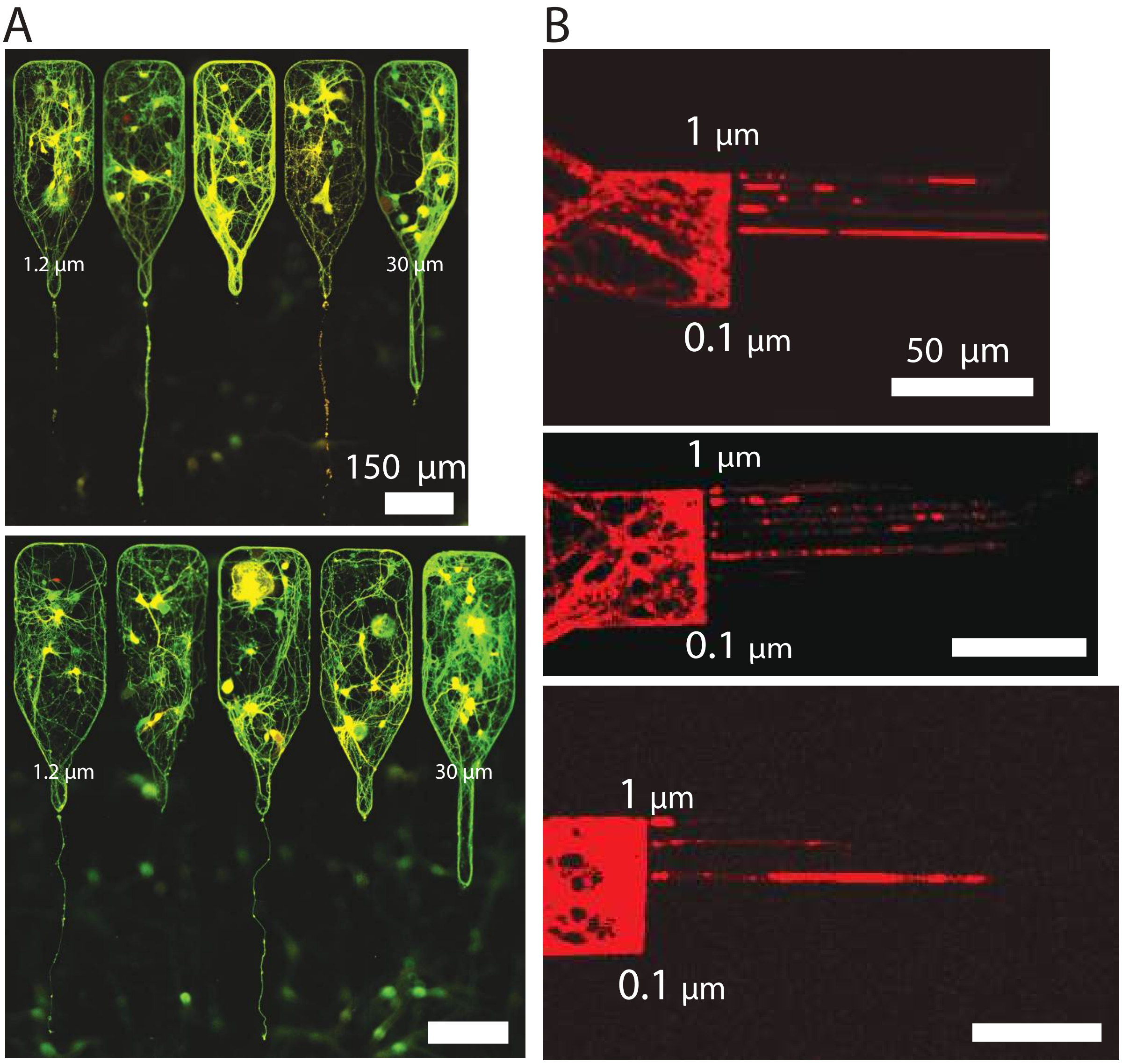

Figure S14: A) Examples of putative single axons penetrating micron tunnels at DIV 12. B) Examples of putative single axons and/or axon branches penetrating submicron tunnels at DIV 10.
